# Anti-Inflammatory Extract from Soil Algae *Chromochloris zofingiensis* Targeting TNFR/NF-κB Signaling at Different Levels

**DOI:** 10.3390/cells11091407

**Published:** 2022-04-21

**Authors:** Peter D. Leitner, Thomas Jakschitz, Ronald Gstir, Stefan Stuppner, Sebastian Perkams, Maren Kruus, Alexander Trockenbacher, Christoph Griesbeck, Günther K. Bonn, Lukas A. Huber, Taras Valovka

**Affiliations:** 1 Institute of Cell Biology, Biocenter, Medical University of Innsbruck, 6020 Innsbruck, Austria; leitner.pd@gmail.com; 2 ADSI—Austrian Drug Screening Institute, Leopold-Franzens University of Innsbruck, 6020 Innsbruck, Austria; thomas.jakschitz@adsi.ac.at (T.J.); ronald.gstir@i-med.ac.at (R.G.); stefan.stuppner@novartis.com (S.S.); 3 Department of Biotechnology and Food Engineering, MCI—The Entrepreneurial School, 6020 Innsbruck, Austria; sebastian@perkams.de (S.P.); maren.kruus@i-med.ac.at (M.K.); alexander.trockenbacher@mci.edu (A.T.); 4 Department of Pediatrics I, Medical University of Innsbruck, 6020 Innsbruck, Austria

**Keywords:** *Chromochloris zofingiensis*, bioactive metabolites, anti-inflammatory effects, NF-κB, mass spectrometry

## Abstract

Inflammatory skin diseases, including atopic dermatitis (AD) and psoriasis, are increasing in populations worldwide. The treatment of patients with AD and other forms of skin inflammation is mainly based on the use of topical corticosteroids or calcineurin inhibitors, which can cause significant side effects with long-term use. Therefore, there is a great need for the development of more effective and less toxic anti-inflammatory agents suitable for the treatment of chronic skin lesions. Here, we screened a number of strains from the ASIB 505 terrestrial algae collection and identified a green algae *Chromochloris zofingiensis* with pronounced anti-inflammatory properties. We found that a crude nonpolar extract of *C. zofingiensis* (ID name NAE_2022C), grown upon nitrogen deprivation, acts as a bioactive substance by inhibiting TNFR/NF-κB responses in human skin keratinocyte HaCaT cells. We also found that NAE_2022C suppressed the secretion of pro-inflammatory cytokine tumor necrosis factor α (TNFα) and several Th1- and Th2-related chemokines in a reconstituted human epidermis. The TNFR/NF-κB pathway analysis showed multiple inhibitory effects at different levels and disclosed a direct targeting of IKKβ by the extract. Bioassay-guided fractionation followed by high-resolution mass spectrometry detected diacylglyceryl-trimethylhomoserine (DGTS), Lyso-DGTS (LDGTS), 5-phenylvaleric acid, theophylline and oleamide as leading metabolites in the active fraction of NAE_2022C. Further analysis identified betaine lipid DGTS (32:0) as one of the active compounds responsible for the NAE_2022C-mediated NF-κB suppression. Overall, this study presents an approach for the isolation, screening, and identification of anti-inflammatory secondary metabolites produced by soil algae.

## 1. Introduction

When properly executed and remedied, the inflammatory process helps to eliminate irritants or pathogens, and promotes tissue repair [1]. However, excessive or persistent inflammation leads to chronic diseases, including autoimmunity, inflammatory skin diseases, and allergies [2,3].

Chronic skin inflammation is normally associated with increased epidermis thickness and the dermal infiltration of immune cells, mainly macrophages, eosinophils, and mast cells. Immune cells produce tumor necrosis factor α, interferon γ (IFNγ), and other pro-inflammatory mediators that stimulate keratinocytes to release a unique profile of chemokines and cytokines, leading to further progression of skin inflammation [4,5,6,7]. The expression of inflammatory mediators mainly depends on the activation of nuclear factor NF-κB, mitogen-activated protein kinases (MAPKs), and activator protein-1 (AP-1), as well as Janus kinase (JAK) and activator of transcription (STAT) pathways [5]. The activation of NF-κB is a central event downstream of tumor necrosis factor receptor (TNFR1/2)-mediated signaling, whereas the STAT pathway is controlled specifically by interferon receptors (IFNAR1/2, IL10R2/IFNLR1 and IFNGR1/2). In addition to glucocorticoids, calcineurin (CNI), and cyclooxygenase inhibitors (NSAIDs), the use of synthetic small molecules targeting inflammatory pathways is a new and promising pharmacological approach to treat inflammatory diseases. Although these drugs effectively inhibit edema, vascular permeability, and other symptoms of skin inflammation, their use is rather limited because of undesirable side effects associated with long-term treatment [8,9,10,11]. As a result, studies have been conducted to analyze the anti-inflammatory effects of natural substances, mainly of plant, fungal and bacterial origin, in order to find more effective and less toxic candidates for topical drugs [12,13,14]. Microalgae and cyanobacteria are also currently receiving a lot of attention from researchers [15].

Microalgae belong to a wide group of eukaryotic and prokaryotic microorganisms that are normally autotrophic, and are present in almost all ecosystems around the globe [15,16,17]. The enormous biodiversity of these microorganisms, and the consequent variability in their biochemical properties, make microalgae cultivations a promising source of novel chemically and biologically active molecules. In particular, the anti-microbial, anti-oxidative and anti-tumor properties of marine algae have been examined for their pharmacological potential [18,19,20]. In contrast to aquatic algal forms, however, aero-terrestrial microalgae remain a largely unexplored niche of new bioactive metabolites. These algae belong to three different evolutionary origins: diatoms (*Bacillariophyceae*, *Ochrophyta*), green algae (*Chlorophyta*, *Streptophyta*), and *Cyanobacteria*, with the majority of organisms belonging to *Chlorophyta* and *Cyanobacteria* [21]. Because of extreme and rapidly changing terrestrial environments, soil algae have developed specific adaptive mechanisms by synthesizing versatile, often species-specific secondary metabolites (SMs). Examples include organic osmolytes such as polyols and betaines, mycosporin-like amino acids (MAAs), pigments (astaxanthin, β-carotene), polyunsaturated fatty acids (PUFAs), and polyphenols among others [22]. This provides a unique opportunity to influence the type of chemical substances produced by soil algae by changing the culture media and conditions. However, there are still a number of challenges related to the isolation and establishment of rare algal strains, cultivation and extraction methods, and the development of screening methods that facilitate the identification of new bioactive compounds and their combinations.

Here, we screened a number of soil algae strains from the unique ASIB 505 microalgae collection for their potential to produce secondary metabolites with anti-inflammatory activities. Among several positive hits, we identified the unicellular green algae *Chromochloris zofingiensis* with pronounced anti-inflammatory properties. We found that the nonpolar extract of *C. zofingiensis*, grown on nitrogen depletion medium, inhibited NF-κB-dependent inflammatory responses in human skin keratinocyte HaCaT cells, as well as in human epidermis equivalents (epiCS) treated with TNFα. The analysis of the TNFR/NF-κB pathway showed multiple inhibitory effects at different levels and revealed a direct targeting of IKKβ by the extract. Finally, by using bioactivity-guided fractionation and mass spectrometry, we identified 1,2-dihexadecanoyl-sn-glycero-3-O-(N,N,N-trimethyl)-homoserine (DGTS), Lyso-DGTS (LDGTS), 5-phenylvaleric acid, theophylline and oleamide as major metabolites in the active fraction of algae extract. Together, our findings point to terrestrial algae as a new natural source of anti-inflammatory compounds and suggest their possible use in the prophylaxis and/or treatment of inflammation, e.g., for inflammatory skin diseases.

## 2. Materials and Methods

### 2.1. Reagents and Antibodies

HPLC and analytical grade methanol, ethanol absolute, and dichloromethane were purchased from Fisher Scientific (Loughborough, UK); formic acid was obtained from ThermoFisher (Life Technologies Austria, Branch Office-Vienna, Austria); dimethyl sulfoxide was purchased from Sigma-Aldrich (St Louis, MO, USA); and Dexamethasone (DM), withaferin A (WFA), and 1,2-dihexadecanoyl-sn-glycero-3-O-(N,N,N-trimethyl)-homoserine (DGTS; C_42_H_81_NO_7_) were purchased from Sigma-Aldrich (Sigma-Aldrich Handels Gmbh, Vienna, Austria). All other chemicals used were of the highest grade of purity commercially available. Recombinant human tumor necrosis factor α, puromycin dihydrochloride, and Hoechst 33342 were provided by ThermoFisher (Life Technologies Austria, Branch Office-Vienna, Austria). Anti-IκBα (L35A5) (#4814S), anti-pIκBα (Ser32) (14D4) (#2859), anti-pSAPK/JNK (#9251), anti-pERK1/2 (#9101), anti-pp38 MAPK (#9211), anti-β-Actin (#4967), and anti-NF-κB p65 (#8242) were obtained from Cell Signaling Technology (Cell Signaling Technology Europe B.V., Frankfurt am Main, Germany), and anti-HA (HA.11) (MMS-101R) was obtained from Biolegend (Biozol Diagnostica Vertrieb GmbH, Eching, Germany). As secondary antibodies, HRP-conjugated goat anti-mouse (A4416) (Sigma-Aldrich Handels Gmbh, Vienna, Austria), HRP-conjugated goat anti-rabbit (A0545) (Sigma-Aldrich Handels Gmbh, Vienna, Austria), anti-rabbit IgG (H + L), F(ab’)2 Fragment Alexa Fluor 488 Conjugate (#4412), and anti-mouse IgG (H + L), F(ab’)2 Fragment Alexa Fluor 594 Conjugate (#8890) (Cell Signaling Technology Europe B.V., Frankfurt am Main, Germany) were used.

### 2.2. Algae Collection

The algae species used in this study originated from the ASIB 505 Culture Collection of Algae [23]. The collection was compiled and provided by Georg Gärtner from the Institute of Botany at the University of Innsbruck. The original ASIB 505 cultures were maintained on Bold’s Basal Medium (BBM) [24] agar slants (10 g L^−1^) in glass test tubes, which were kept at the optimal temperature of 13 °C and at a light intensity of 300–3000 lux under cool-white fluorescent lamps on a 12 h day–night rhythm. Strains were transferred every six months to fresh media.

### 2.3. Algae Cultivation and Stress Conditions

Algae were cultivated by spinning at 100 rpm in BBM medium at 25 °C using 16:8 h day–night rhythm. Cultures were grown at a light intensity of 200 µE m^−2^ s^−1^ for several days. These pre-cultures were used for large-scale cultivation in 1000 mL bottles. Cell cultures were adjusted to an optical density of 0.3 at 750 nm and grown under normal or various stress conditions, including nitrogen deprivation, high salinity (0.2 M NaCl), and high glucose concentration (5%).

### 2.4. Preparation of Algae Extracts

The lyophilized 100 mg algae biomass was extracted with 4 mL of ethanol:water 8:2 (*v*/*v*) or dichloromethane:methanol 1:1 (*v*/*v*) solvents by vortexing with 1 mg of Ø 0.25–0.5 mm glass beads (A553.1) (Carl Roth GmbH + Co. KG, Karlsruhe, Germany) for 3 min at RT. The extracts were centrifuged at 5000 g for 15 min at RT, and supernatants were collected. The procedure was repeated at least 3 times. The supernatants were pooled and lyophilized at RT using Thermo Scientific Savant SPD131 DDA SpeedVac Concentrator (Fisher Scientific GmbH, Schwerte, Germany). Dry extracts were stored at −20 °C until further use. Lyophilized extracts were dissolved in 100% DMSO to a final concentration of 10 mg mL^−1^, sterilized by filtration, and stored in 1 mL aliquots at −20 °C, protected from light.

### 2.5. Cell Culture and Treatment

The human keratinocyte cell line HaCaT (#T0020001) (AddexBio, San Diego, CA, USA) and human embryonic kidney HEK 293T cells (ATCC #CRL-1573) (American Type Culture Collection, Rockville, MD, USA) were cultured in Dulbecco’s Modified Eagle Medium (DMEM; D6429) (Sigma-Aldrich Handels Gmbh, Vienna, Austria) supplemented with 10% (*v*/*v*) FBS (ThermoFisher, Life Tecnologies Austria, Branch Office-Vienna, Austria) and antibiotics (100 U mL^−1^ penicillin and 100 μg mL^−1^ streptomycin) (P0781 Sigma-Aldrich Handels Gmbh, Vienna, Austria) at 37 °C, 5% CO_2_, and 95% relative humidity, and routinely tested for the absence of mycoplasma.

For anti-inflammatory activity screening and pathway analyses, HaCaT cells were either irradiated with 0.15 J/cm^2^ UVB or stimulated with 0.75 ng mL^−1^ TNFα in a fresh DMEM medium containing 2% (*v*/*v*) FBS for the indicated times. The stimulus doses used resulted in a several-fold increase in NF-κB activity without substantially decreasing (<10%) cell viability. When specified, cells were pre-treated for 1 h with 100 nM DM, 750 nM WFA, or indicated non-cytotoxic concentrations of algae extracts, chromatography fractions, and DGTS.

### 2.6. Generation of NF-κB Reporter HaCaT Cell Line

The retroviral plasmid pHR CMV Luc Sin-18 [25] was used to generate the NF-κB reporter construct. For this, the human cytomegalovirus (CMV) promoter was replaced with the minimal pTA promoter fused to four κB binding sites (5′-GGGAATTTCC-3′) and a linker sequence using the ClaI and BamHI sites. To facilitate the selection of the transduced cells, the antibiotic resistance gene *pac*, controlled by the pSV40 promoter, was inserted immediately downstream of the firefly luciferase coding sequence. The resulting pHR NF-κB/pTA Luc SIN-18 plasmid was sequence verified.

To produce viruses, 4 × 10^6^ HEK 293T cells were transfected with 3.5 μg of the envelope pMD.G plasmid, 6.5 μg of the packaging psPAX2 plasmid, and 10 μg of pHR NF-κB/pTA Luc SIN-18 by calcium phosphate co-precipitation. After 36 h, the viral supernatants were collected, concentrated with Retro-X Concentrator (Clontech, Takara Bio USA Inc., Mountain View, CA, USA), and used to infect HaCaT cells. The selection was carried out for 4 weeks in DMEM supplemented with 10% (*v*/*v*) FBS, 100 U mL^−1^ penicillin and 100 μg mL^−1^ streptomycin, and 2 µg mL^−1^ puromycin.

### 2.7. Reconstituted Human Epidermis Model

Human epidermal equivalents (epiCS) (Cell Systems GmbH, Troisdorf, Germany) were cultured on polycarbonate filter inserts at the air-liquid interface using epiCS culture medium (#CS-3051) (Cell Systems GmbH, Troisdorf, Germany) at 37 °C, 5% CO_2_, and 95% relative humidity. Cytokine and chemokine release was analyzed in epiCS subnatants using Human Cytokine Magnetic 25-Plex Panel (#LC2005) (Invitrogen, Life Technologies Austria, Branch Office-Vienna, Austria) according to the manufacturer’s protocol. Measurements were performed using the Luminex FleXmap 3D Instrument System (R&D Systems, Minneapolis, MN, USA).

### 2.8. Cell Viability Assays

The cytotoxicity of algae extracts was evaluated using the Resazurin Cell Viability Kit (Cell Signaling Technology Europe B.V., Frankfurt am Main, Germany) and CytoTox 96 Non-Radioactive Cytotoxicity Assay (Promega GmbH, Walldorf, Austria) according to the manufacturers’ protocols. Briefly, after attaching to the plate, HaCaT cells were treated with different concentrations (0.1–100 µg mL^−1^) of extract and incubated for 24 h. Fluorescence for the resazurin assay and the absorbance for LDH release were measured with a micro plate reader (Tecan Spark, Männedorf, Switzerland) at excitation/emission wavelengths of 540 nm/590 nm and 490 nm, respectively.

### 2.9. Luciferase Reporter Assay

NF-κB reporter HaCaT cells were grown overnight, pre-treated for 1 h with the indicated concentrations of algae extracts or inhibitors, and irradiated with 0.15 J/cm^2^ UVB or stimulated with TNFα, as specified. After treatment, cells were washed with ice cold 1× phosphate-buffered saline (PBS) and extracted with Lysis buffer (250 mM Tris-HCl pH 7.5, 2% Triton X-100) for 5 min at RT. Then, 40 µL of lysates was mixed with 150 µL of Luciferase Assay buffer (25 mM glycylglycine pH 7.8, 5 mM ATP, 15 mM MgSO_4_), injected with 100 µL of Luciferase Substrate buffer (20 mM glycylglycine pH 7.8, 40 mM (4*S*)-2-(6-hydroxy-1,3-benzothiazol-2-yl)-4,5-dihydrothiazole-4-carboxylic acid), and the luminescence was measured using a micro plate reader (Mithras LB940) (Berthold Technologies LLC, Bad Wildbad, Germany). The relative luciferase activities were normalized to protein concentrations.

### 2.10. High-Performance Liquid Chromatography (HPLC) and Mass Spectrometry Analysis

The fractionation of extracts was performed by size-exclusion chromatography on Kinetex 5 µm C18 100A 250 × 10 mm column (Agilent Technologies, Santa Clara, CA, USA) using a Waters Aquity Arc LC System equipped with a photodiode array (PDA 200–800 nm) (Waters Corp., Milford, MA, USA). The mobile phase was a mixture of 0.1% formic acid in water (solvent A) and acetonitrile (solvent B) at a flow rate of 3 mL min^−1^. The gradient profile was as follows: 24 min from 10% B to 90% B; 30 min 10% B. The injection volume of the samples was 0.5 mL. The mass measurement was performed in a range of 100–800 *m*/*z* by using a Waters QDA single-quad mass spectrometer (Waters Corp., Milford, MA, USA) in electro-spray positive and negative modes.

The components of NAE fractions were analyzed by a MaXis Impact HR-qTOF Mass Spectrometer (Bruker, Bremen, Germany) coupled to an UHPLC system (Thermo Fisher Scientific, Waltham, MA, USA) with an Agilent Zorbax RRHD Eclipse Plus C18 100 × 2.1 mm column (Agilent Technologies, Santa Clara, CA, USA). The gradient program was used with the mobile phase, combining 0.1% formic acid in water (solvent A) and acetonitrile (solvent B) as follows: 16 min from 50% to 90% B; 2 min from 90% to 90% B. The parameters were as follows: flow rate, 0.5 mL min^−1^; injection volume, 10 µL.

The MaXis Impact mass spectrometer was operated in the data-dependent mode, selecting the top 12 most abundant isotope patterns with charge > 1 from the survey scan with an isolation window of 1.6 mass-to-charge ratio (*m*/*z*). Survey full-scan MS spectra were acquired from 300 to 1750 *m*/*z* at a resolution of 40,000 with a maximum injection time (IT) of 120 ms and an automatic gain control target (AGC) of 1 × 10^6^. The selected isotope patterns were fragmented by higher-energy collisional dissociation with a normalized collision energy of 28 at a resolution of 35,000 with a maximum IT of 120 ms, and an AGC target of 5 × 10^5^. The identification of compounds was based on the assignment of the molecular ions observed in MS spectra, typical retention time, mass accuracy, and MS/MS spectra interpretation. The data were analyzed using Progenesis QI software (Waters Corp., Milford, MA, USA) and the NIST 05 MS library (f-fit > 700; r-fit > 650) with the indicated parameters.

### 2.11. Microscopy

HaCaT cells were seeded at a density of 17,000 cells per well in a black 96-well plate (#3603) (Corning Costar, NY, USA) and incubated at 37 °C and 5% CO_2_ for 24 h. Medium was exchanged for a fresh one containing 2% (*v*/*v*) FBS and cells were stimulated for 20 min with 10 ng mL^−1^ TNFα in the presence of 0.1% (*v*/*v*) DMSO, 10 µg mL^−1^ NAE_2022C, or 750 nM WFA. After treatment, cells were fixed with 4% (*v*/*v*) formaldehyde in PBS for 15 min at RT, washed three times with PBS, and incubated with Blocking buffer (1× PBS, 5% (*v*/*v*) BSA, 0.3% (*v*/*v*) Triton X-100) for 1 h at RT. For the immunofluorescent detection of proteins, cells were incubated with primary (1:1000) antibodies followed by Alexa Fluor 594- and Alexa Fluor 488-conjugated secondary antibodies (1:1000). DNA was stained with a solution of PBS containing 1 ng mL^−1^ Hoechst 33342 for 5 min at RT. Cells were washed three times for approx. 5 min with PBS and the plates were then analyzed using an ImageXpress Micro XLS microscope and MetaXpress software (Molecular Devices, San Jose, CA, USA).

Algae cultures were analyzed by bright field microscopy using an Eclipse 50i Microscope (Nikon, Tokio, Japan).

### 2.12. Immunoblotting

Cells were washed with 1× PBS and lysed in ice-cold Nonidet P40 lysis buffer (40 mM Tris-HCl pH 8.0, 100 mM NaCl, 0.5% (*v*/*v*) Nonidet-P40, 10 mM β-glycerophosphate, 10 mM NaF, 1 mM EDTA, 1 mM PMSF, 1 μg/mL pepstatin, 1 μg/mL aprotinin, and 1 μg/mL leupeptin) for 10 min on ice. Upon centrifugation at 10,000× *g* and 4 °C, cleared lysates were normalized, resolved by SDS-PAGE, transferred onto a nitrocellulose membrane (Amersham Protran Premium 0.2 µm NC, Global Life Sciences Solutions Operations UK Ltd., Little Chalfont, UK), and immunoprobed with the antibodies indicated according to manufacturer’s recommendations. Enhanced chemiluminescence detection (Western Bright ECL, Advansta, Biozym Scientific GmbH, Oldendorf, Germany) was used in immunoblot assays.

### 2.13. In Vitro IKK Kinase Assay

The pcDNA 3.1(+) HA-IKKβ plasmid was generated by cloning the coding region of IKKβ HA tagged at the N-terminus into the pcDNA 3.1(+) vector (Invitrogen, Life Tecnologies Austria, Branch Office-Vienna, Austria). HEK 293T cells were transfected with the HA-IKKβ-expressing DNA construct using calcium phosphate precipitation. After 24 h, cells were washed with ice-cold PBS and lysates were prepared as described above. Three hundred nanograms of cell lysates were incubated with 2 µg of anti-HA (HA.11) antibody for 2 h, followed by 30 µL of 50% slurry protein A sepharose (GE Healthcare Bio-Sciences AB, Uppsala, Sweden) for another 1 h or overnight at 4 °C. Immunoprecipitates were washed three times with lysis buffer followed by a single wash with a high-salt buffer containing 500 mM NaCl, and then with kinase buffer without ATP. The kinase assay was performed in 30 μL of kinase buffer containing 50 mM HEPES pH 7.5, 10 mM MgCl_2_, 1 mM dithiothreitol, 10 mM β-glycerophosphate, 1 mM ATP, 8 μCi of [γ-^32^p] ATP and varying amounts of 2022C extract (0.13, 0.23 and 0.46 µg µL^−1^) or DMSO. Two micrograms of recombinant HIS-IκBα purified from *Escherichia coli* strain BL21 (DE3) CodonPlus-RIL (Agilent Technologies, Santa Clara, CA, USA) by affinity chromatography were used as substrate. Reactions were stopped by adding a 5× SDS-PAGE sample buffer, followed by heating at 95 °C for 5 min. Proteins were separated by 10% (*wt*/*vol*) SDS-PAGE and subjected to autoradiography.

### 2.14. Statistical Analysis

Data are presented as mean ± SD values. Statistical comparisons were calculated using a two-sample Student’s *t*-test. *p* values are denoted as follows: * *p* < 0.05, ** *p* < 0.01, and *** *p* < 0.001. To evaluate the anti-inflammatory effects of the tested extracts, the effect size calculation with a Cohen’s d value cut off ≥ 1 was used.

## 3. Results

### 3.1. Anti-Inflammatory Activity Screening of Soil Algae Extracts

**Figure 1:**
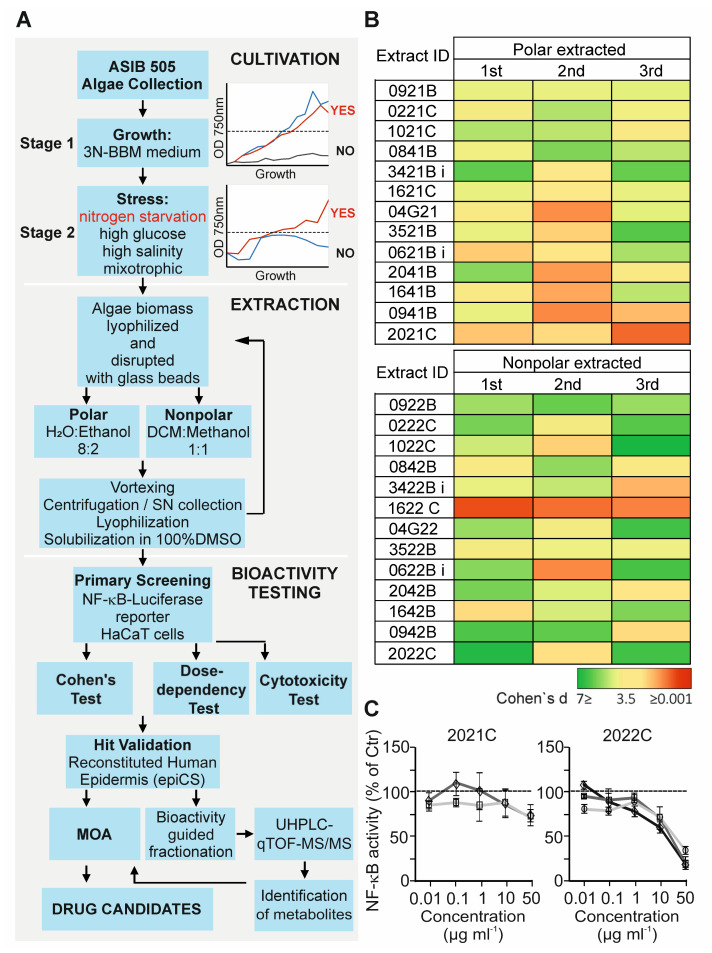
Anti-inflammatory activity screening of algae extracts. (**A**) Workflow of the screening procedure for the identification of anti-inflammatory metabolites from soil algae. In the cultivation box, the growth curves represent examples of selected strains cultivated under standard conditions (Upper) or nitrogen deprivation (Lower); DCM, dichloromethane; MOA, mode of action. (**B**) Heatmap of effect sizes (Cohen’s d, with higher value indicating repression and lower value indicating increase in reporter activity) of NF-κB reporter inhibition by algae extracts in TNFα-stimulated HaCaT cells. Biological replicates are named 1st, 2nd, and 3rd, and are presented as the mean of the technical replicates (*n* = 3). (**C**) Dose-dependent anti-inflammatory effects of polar (2021C) and nonpolar (2022C) *C. zofingiensis* extracts in HaCaT cells. NF-κB activity in DMSO-treated cells is taken as 100%. Each curve represents an independent experiment. Values are mean ± SD of technical replicates (*n* = 3); Ctr, control (DMSO + TNFα).

To reveal the novel inhibitors of inflammation, we selected thirty-six algae strains from the ASIB 505 microalgae collection (Table S1) based on their ability to grow in liquid culture (stage 1) and efficiently adapt to various administrated stress conditions (stage 2), i.e., nitrogen deprivation, glucose, high salinity, and mixotrophic stresses (Figure 1A). In this two-stage cultivation procedure, forty-nine algae biomass samples were obtained and subsequently extracted with nonpolar (1:1 dichloromethane:methanol) and polar (8:2 ethanol:water) solvents, resulting in a total of ninety-eight extracts. Prior to bioactivity testing, the extracts were evaluated for their cytotoxicity by measuring metabolic activity and lactate dehydrogenase (LDH) release using human skin keratinocyte HaCaT cells. We found that all extracts tested decreased cell viability by less than 10% when concentrations of less than 100 µg mL^−1^ were used (Table S2).

**Figure 2:**
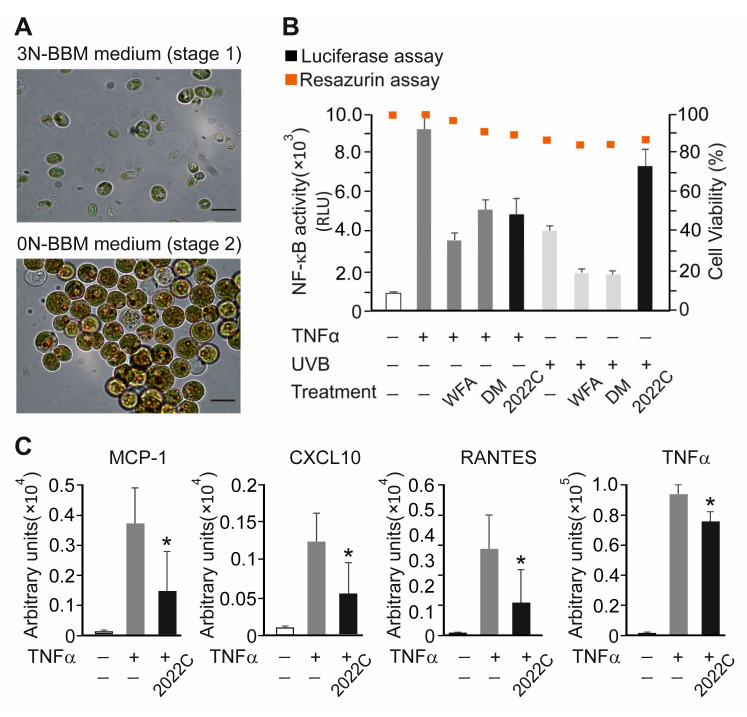
Inhibitory effects of NAE_2022C on TNFα- and UVB-induced inflammatory responses. (**A**) Brightfield micrographs of *C. zofingiensis* cultivated at standard growth conditions (stage 1; Upper) and after nitrogen deprivation stress (stage 2; Lower). Scale bar, 10 μm. (**B**) NAE_2022C inhibited TNFα- but not UVB-induced NF-κB responses. HaCaT cells were incubated with 100 nM DM, 750 nM WFA or 10 µg mL^−1^ NAE_2022C for 1 h and subsequently stimulated with 0.75 ng mL^−1^ TNFα or irradiated with 0.15 J/cm^2^ UVB. The luciferase activity was measured in cell lysates 6 or 24 h post-stimulation, respectively (left y-axis). The cell viability was evaluated using a resazurin assay and shown as orange squares (right y-axis). Data are presented as mean ± SD (*n* = 3). (**C**) The suppression of TNFα-induced pro-inflammatory mediators in reconstituted human epidermis (epiCS) by NAE_2022C. The release of cytokines and chemokines was determined in the medium after the treatment of epiCS with 0.75 ng mL^−1^ TNFα for 24 h (MCP1, RANTES and TNFα) and 48 h (CXCL10) using the Luminex Assay. Data represent the mean ± SD of at least three independent biological replicates. * *p* < 0.05 vs. samples treated with DMSO and TNFα.

To assess the anti-inflammatory activity of extracts, we tested their ability to inhibit a TNFα-induced NF-κB-dependent luciferase reporter expressed stably in HaCaT cells. This cell line represents a common test system for functional studies of keratinocytes in chronic skin diseases and for studies of anti-inflammatory substances. The cells were stimulated with 0.75 ng mL^−1^ TNFα for 6 h in the presence of 0.1% DMSO or 10 µg mL^−1^ of polar (PAE) or nonpolar (NAE) algae extracts, and the luciferase activity was compared in cell lysates. To reduce the incidence of false positive hits, the inhibition of the reporter was verified by the effect size calculation with a Cohen’s d value (measure of effect size in the context of analysis of variance) cut off ≥ 1. As shown in Figure 1B, of the thirty-six strains tested, thirteen exhibited inhibitory activities which could be extracted either with polar, nonpolar or both solvents. The selected extracts from the primary screening were further validated over a broader concentration range (0.01 to 50 µg mL^−1^). Finally, six extracts were confirmed as positive hits based on their dose-dependent effects with the inhibition rate of ≥30% at 50 µg mL^−1^ (Figure 1C and Figure S1). Among them, the NAE with ID name 2022C was chosen for further investigation because of its high potency to inhibit NF-κB activity in response to TNFα (approx. 75% at 50 µg mL^−1^). The extract originates from a strain with the ID name V142 represented by the algae species *Chromochloris zofingiensis* (Phylum: *Chlorophyta*; Subphylum: *Chlorophyta*; Class: *Chlorophyceae*; Order: *Sphaeropleales*; Family: *Chromochloridaceae*; Genus: *Chromochloris*) cultivated under nitrogen starvation (Figure 2A). Interestingly, the PAE of *C. zofingiensis* (ID_2021C) was less capable of inhibiting NF-κB response (approx. 30% at 50 µg mL^−1^), suggesting a predominantly hydrophobic nature of extracted anti-inflammatory metabolites (Figure 1C).

Next, we compared the efficacy of extract 2022C (NAE_2022C) to that of known anti-inflammatory, synthetic, and natural NF-κB-inhibiting drugs, namely dexamethasone and withaferin A [26,27,28]. The reporter activity was reduced by approx. 42% (1.7-fold) and 64% (2.7-fold) in HaCaT cells treated with 0.75 ng mL^−1^ TNFα when 100 nM DM and 750 nM WFA were used, respectively. The extent of inhibition produced by NAE_2022C (10 µg mL^−1^) was approx. 48% (1.9-fold), indicating its potential to interfere with cytokine receptor-mediated NF-κB activation. The viability of cells, determined 24 h post-treatment, was higher than 80% in all test conditions (Figure 2B). We also investigated whether NAE_2022C implemented a more general NF-κB inhibition, similarly to DM and WFA, or if it acted specifically, e.g., in a stimulus-specific manner. For this purpose, we analyzed NF-κB reporter activity in HaCaT cells irradiated with 0.15 J/cm^2^ UVB and treated with DM, WFA and NAE_2022C (Figure 2B). As expected, DM and WFA markedly decreased UVB-induced NF-κB activity, resulting in approx. 50% inhibition. In contrast, the NAE_2022C treatment did not inhibit, but rather enhanced, the activation of NF-κB by UVB light. We conclude that NAE_2022C acts differently than broad-range NF-κB inhibitors, suggesting its distinct mode of action.

### 3.2. NAE_2022C Inhibits TNFα-Induced Pro-Inflammatory Mediators in Reconstituted Human Epidermis

We next examined the anti-inflammatory activity of NAE_2022C by measuring cytokine and chemokine secretion in a reconstituted human epidermis model. Human epidermal equivalents (epiCS) were incubated with 10 µg mL^−1^ NAE_2022C added to the cultivation medium. The level of mediators in the medium was determined after the treatment of epiCS with 0.75 ng mL^−1^ TNFα for 24 and 48 h. As shown in Figure 2C, TNFα induced a significant increase in the protein levels of monocyte chemotactic protein-1 (MCP-1; 27-fold), C-X-C motif chemokine ligand *10* (*CXCL10*; 12-fold), chemokine (C-C Motif) ligand 5 (RANTES; 78-fold) and tumor necrosis factor alpha (TNFα; 87-fold), compared with those in the control. When epiCs were treated with NAE_2022C, we detected a decrease in the secretion of MCP1, CXCL10, RANTES and TNFα without detectable cytotoxic effects. The results indicate that NAE_2022C exerts anti-inflammatory activity in skin-relevant settings by interfering with TNFα-mediated responses.

### 3.3. Nuclear Translocation of NF-κB and IκBα Degradation Are Suppressed by NAE_2022C

**Figure 3:**
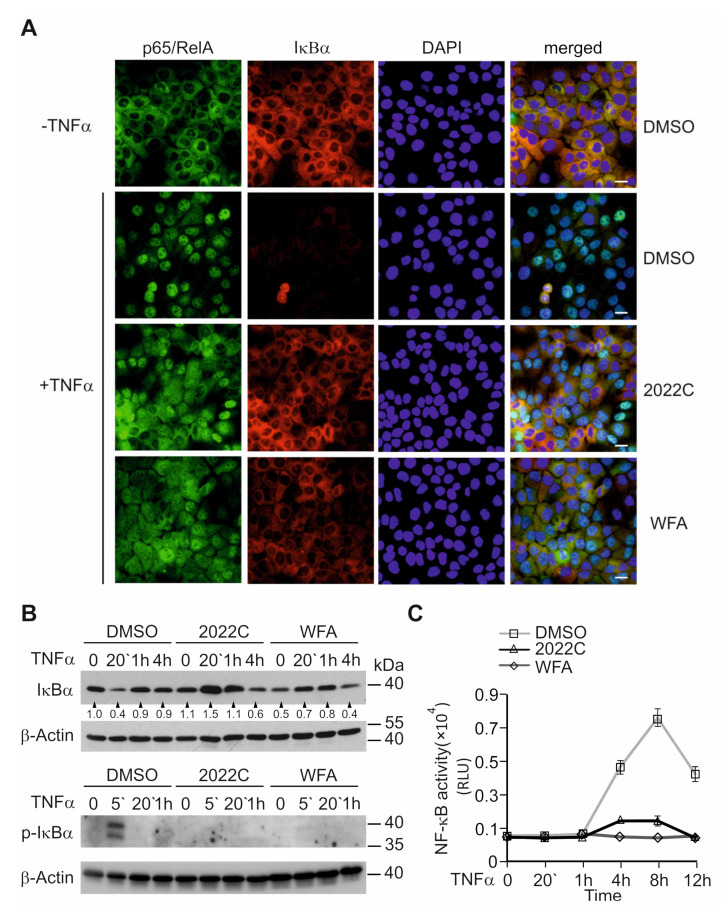
Effects of NAE_2022C on IκBα degradation and the nuclear translocation of NF-κB in TNFα-stimulated HaCaT cells. (**A**) NAE_2022C inhibits the nuclear accumulation of p65/RelA in response to TNFα. Cells were either left untreated or stimulated with 10 ng mL^−1^ TNFα for 20 min in the presence of 0.1% DMSO, 750 nM WFA, or 10 µg mL^−1^ NAE_2022C. The p65/RelA (green) and IκBα (red) proteins were visualized by indirect immunofluorescence. Hoechst (blue) was used to localize nuclei. Scale bar, 10 µm. (**B**) The effects of WFA and NAE_2022C on the degradation and re-synthesis of IκBα in response to TNFα. NF-κB-reporter HaCaT cells were incubated with DMSO, WFA or NAE_2022C, as described in (**A**), and then treated with 0.75 ng mL^−1^ TNFα for the indicated times. Whole-cell lysates were analyzed by immunoblotting using IκBα, phospho-IκBα and β-Actin-specific antibodies. The intensity of the bands was quantified using Image J normalized to β-Actin, and denoted as the fold change relative to the control. (**C**) NF-κB responses were evaluated in cell lysates from (**B**) by measuring luciferase activity. Values are means ± SD of at least three measurements.

To understand the anti-inflammatory effects of NAE_2022C, we explored the NF-κB inhibition in detail, in the context of TNFα signaling. In this pathway, NF-κB is activated through the IκB kinase beta (IKKβ)-mediated phosphorylation and subsequent degradation of inhibitory κB (IκB) proteins. This leads to the accumulation of free NF-κB dimers in the nucleus where they bind to κB DNA consensus sequences and transactivate genes [29,30]. Initially, we looked at the effects of NAE_2022C on the TNFα-induced nuclear translocation of the p65/RelA subunit of NF-κB. Immunofluorescence staining for p65/RelA and IκBα revealed that both proteins were mainly cytoplasmic in unstimulated HaCaT cells (Figure 3A). The treatment of cells with 10 ng mL^−1^ TNFα for 20 min resulted in, predominantly, the nuclear staining of p65/RelA concomitant with a decrease in IκBα signals. Interestingly, we found that both WFA and NAE_2022C decreased the TNFα-induced nuclear accumulation of NF-κB and retained cytoplasmic IκBα staining. We assumed that, similar to WFA, NAE_2022C inhibited the nuclear translocation of NF-κB by preventing IκBα degradation. To verify this, we analyzed the effects of WFA and NAE_2022C on the kinetics of IκBα degradation and re-synthesis in HaCaT cells treated with TNFα. As shown in Figure 3B, TNFα initiated the rapid and transient degradation of IκBα, with the re-synthesis of IκBα occurring by 1 h post-stimulation. Treatment with both WFA and NAE_2022C markedly repressed the loss of IκBα in response to TNFα. These results were consistent with the inhibition of the NF-κB reporter activity observed in WFA- and NAE_2022C-treated cells (Figure 3C). Hence, we conclude that NAE_2022C inhibits NF-κB responses by interfering with IκBα degradation and the nuclear translocation of this transcription factor.

### 3.4. NAE_2022C Inhibits the IKK-Dependent Phosphorylation of IκBα and ERK1/2 Downstream of TAK1

**Figure 4:**
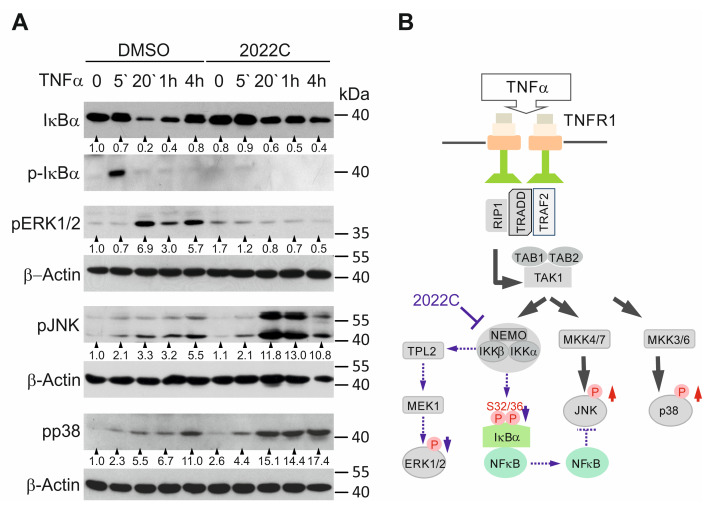
NAE_2022C inhibits TNFα-induced NF-κB and ERK1/2 activation by targeting the IKK complex. (**A**) HaCaT cells were stimulated with 0.75 ng mL^−1^ TNFα for different times, as indicated in the presence or absence of 10 µg mL^−1^ NAE_2022C. Cells were analyzed by immunoblotting using the indicated antibodies. The intensity of the bands was analyzed using Image J, as in Figure 3B. (**B**) Schematic representation of the TNFR/NF-κB pathway. Inhibition of the IKK complex by NAE_2022C is indicated. Blue and red arrows represent a decrease and increase in protein phosphorylation, respectively. Dashed arrows indicate the NAE_2022C-mediated inhibition of signaling events downstream of IKKs.

Because the phosphorylation of IκBα at Ser32 and Ser36 is critical to trigger its ubiquitin-dependent 26S-proteasomal degradation [31,32], we first evaluated the effects of NAE_2022C on IκBα phosphorylation induced by TNFα in HaCaT cells. As expected, we detected a transient IκBα phosphorylation after 5 min of TNFα stimulation, which preceded the IκBα degradation in the control cells (Figure 4A). Pre-treatment with 750 nM WFA or 10 µg mL^−1^ NAE_2022C markedly inhibited TNFα-induced IκBα phosphorylation and degradation, suggesting that NAE_2022C acted upstream of IκBα in the TNFR/NF-κB pathway (Figure 3B and Figure 4A). We also observed a remarkable inhibition of TNFα-induced ERK1/2 phosphorylation upon treatment with NAE_2022C. The phosphorylation of IκBα was directly mediated by the IKKβ subunit of the IKKα/β/NEMO complex, whereas Tpl2 kinase and its target MEK1, both acting downstream of IKKα/β/NEMO, controlled ERK1/2 activation in response to TNFα (Figure 4B). Since the IKK complex is an essential component of these pathways [32,33], we suppose that NAE_2022C may work at the level of, or upstream of, IKKs. To dissect this, we additionally investigated the phosphorylation of JNK and p38. Although JNK and p38 kinases share TAK1 as a key upstream regulator with ERK1/2 and NF-κB, they are not activated by IKKs. Figure 4A indicates that the treatment with TNFα alone induced JNK and p38 phosphorylation in the HaCaT cells. Interestingly, NAE_2022C did not hinder the TAK1-mediated activation of JNK and p38 in response to TNFα, and even enhanced it. Therefore, we conclude that NAE_2022C manifests its inhibitory effects by suppressing IKKs.

### 3.5. NAE_2022C Directly Inhibits IKKβ Kinase Activity

**Figure 5:**
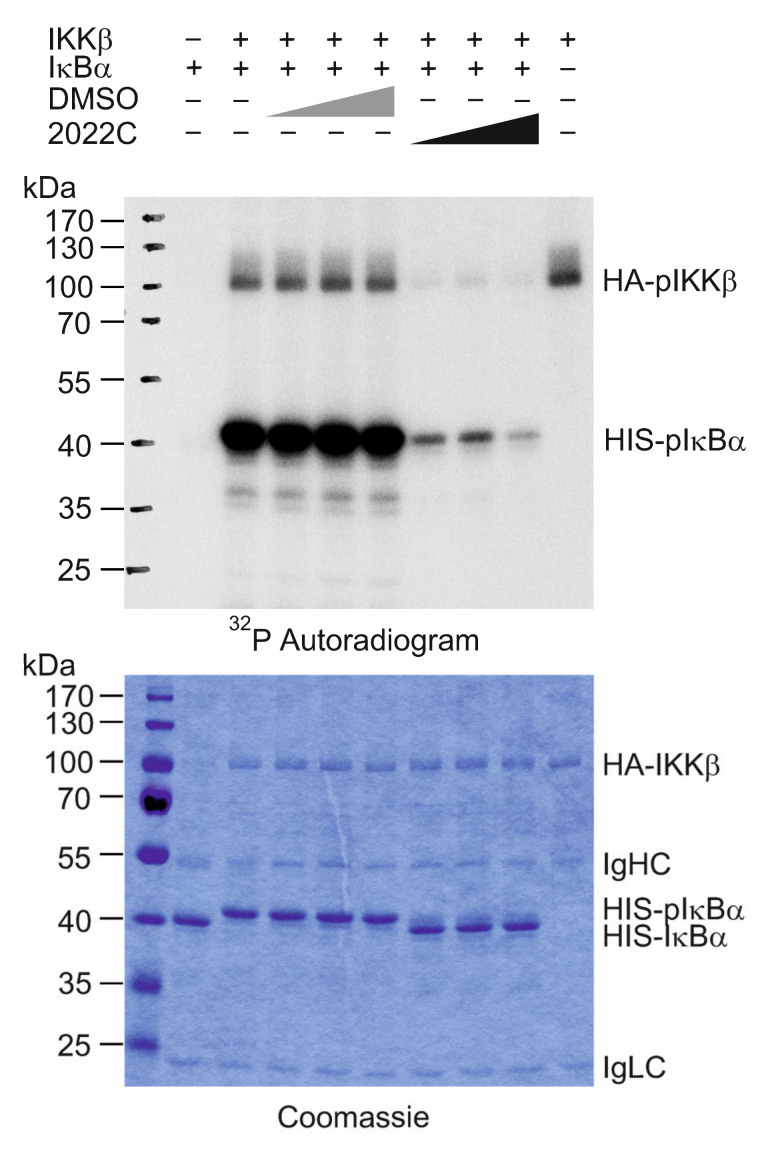
NAE_2022C directly inhibits IKKβ kinase activity. IKKβ activity was analysed by in vitro kinase assay using recombinant HIS-IκBα and [γ-^32^P]ATP in the presence of various doses of NAE_2022C (0.13, 0.23 and 0.46 µg µL^−1^). The reactions were resolved on SDS-PAGE and analyzed by autoradiography; IgHC, immunoglobulin heavy chain; IgLC, immunoglobulin light chain.

Next, we examined how NAE_2022C affects the activity of the IKK complex in response to TNFα. For this, we ectopically expressed HA-tagged IKKβ in HEK 293T cells and evaluated its kinase activity using an in vitro immunocomplex kinase assay. As shown in Figure 5, NAE_2022C dose-dependently inhibited the phosphorylation of recombinant HIS-IκBα used as a substrate. Moreover, we found that the 2022C extract prevented the autophosphorylation of IKKβ, suggesting a direct inhibition of the intrinsic kinase activity rather than interference with the substrate recognition or binding.

### 3.6. Bioactivity-Guided Fractionation of NAE_2022C and the Identification of Representative Metabolites

**Figure 6:**
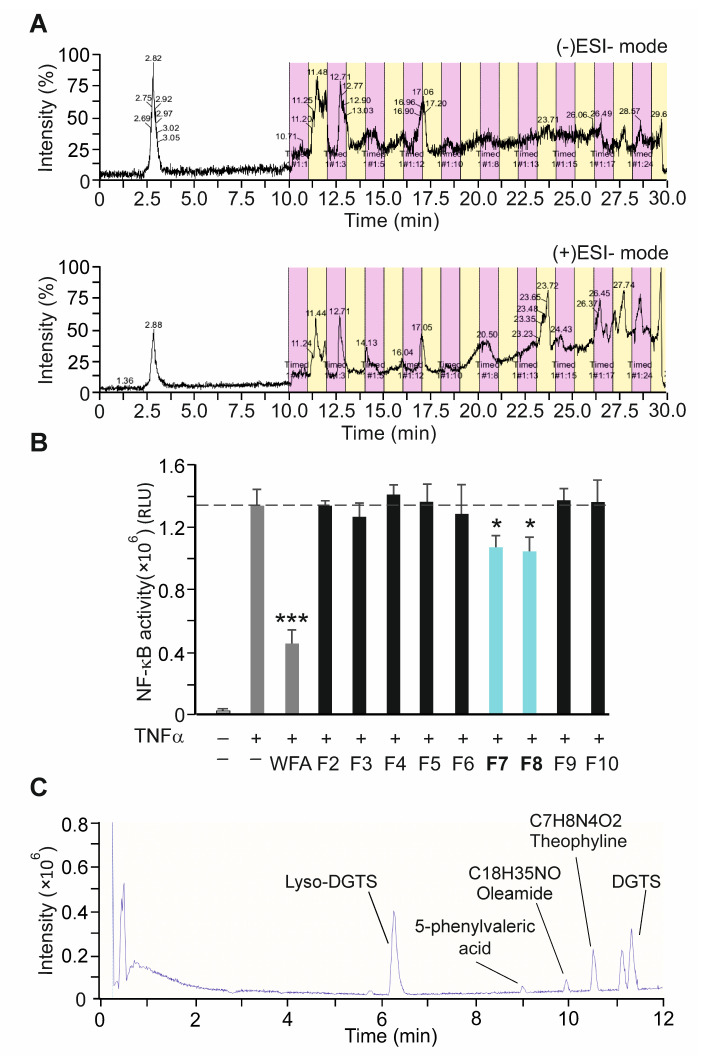
Bioactivity-guided fractionation of NAE_2022C and the detection of major metabolites. (**A**) Total ion HPLC-MS chromatograms of crude 2022C extract. Colored bars indicate collected fractions and corresponding peaks in positive and negative (−)ESI and (+)ESI MS ionization modes. (**B**) The bioactivity of NAE_2022C fractions (F2–F10; 1 µg mL^−1^) were analyzed with the NF-κB reporter cell-based assay, as shown in Figure 2B. Data represent means ± SD of three independent biological triplicates. * *p* < 0.05; *** *p* < 0.001 vs. samples treated with DMSO and TNFα. (**C**) UHPLC and mass spectrometry analysis of NAE_2022C active fraction (F7 + F8). Identified molecules and corresponding chromatogram peaks are indicated.

To identify candidate compounds responsible for specific anti-inflammatory effects, NAE_2022C was fractionated using preparative HPLC coupled to high-resolution mass spectrometry. Twenty fractions (F1–20), retaining different intensity peaks in both positive (+)ESI and negative (−)ESI ion modes, were obtained and analyzed with the NF-κB reporter cell-based assay (Figure 6A,B). Among all fractions tested, only F7 and F8 induced a more than 20% inhibition of NF-κB reporter activity when used at the concentration of 1 µg mL^−1^. Both fractions shared a mutual peak with a retention time of 17.05 min in the (+)ESI mode and 17.06 min in the (−)ESI mode. Furthermore, F7 and F8 showed comparable (+)ESI ion spectra when analyzed using an ultra-high-performance liquid chromatography-quadrupole time-of-flight mass spectrometry (UHPLC-qTOF-MS/MS) system (Figure 6C). The identification of the detected molecules was performed by comparing the mass spectrometry data with the NIST 05 MS library (f-fit > 700; r-fit > 650) [34]. We identified several major metabolites in the combined active fraction (F7 + F8) which were assigned as DGTS (712.61 *m*/*z* and 740.64 *m*/*z*), LDGTS (474.38 *m*/*z*), 5-phenylvaleric acid (179.10 *m*/*z*), oleamide (282,28 *m*/*z*), and theophylline (181.08 *m*/*z*) (Figure 6C and Table S3). We also showed that the active fraction (F7 + F8) efficiently inhibited IKKβ kinase activity in a similar way to that observed for NAE_2022C (Figure S2). We conclude that the active fraction contains the compound(s) responsible for the specific anti-inflammatory activities of NAE_2022C, including the inhibition of the IKK complex.

### 3.7. The Betaine Lipid DGTS Is an Anti-Inflammatory Metabolite of C. zofingiensis Inhibiting NF-κB

**Figure 7:**
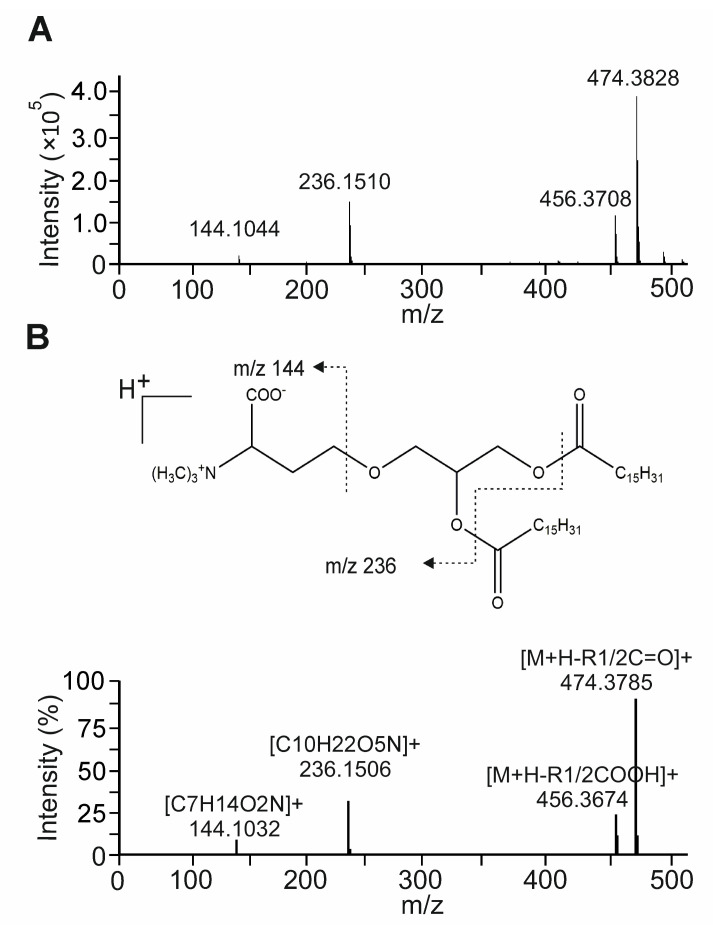
MS/MS analysis of DGTS species identified in NAE_2022C. (**A**) Representative MS/MS spectrum of [M + H]^+^ ion at *m*/*z* 740.6 of DGTS identified in the active fraction (F7 + F8) of NAE_2022C. (**B**) MS/MS spectrum and fragmentation pattern of [M + H]^+^ ion at *m*/*z* 712.6 of DGTS (16:0–16:0) standard [35,36].

We next characterized, in detail, one of the most abundant metabolites identified in fraction F7 and F8: a glycolipid from the class of diacylglycerol-trimethylhomoserine (DGTS). The structural features of the DGTS molecular species (712.61 *m*/*z* and 740.64 *m*/*z*) detected were confirmed through the identification of the typical product ions and fragmentation patterns observed in the MS/MS spectra. The representative (+)ESI-MS/MS spectrum of DGTS showed the typical reported ion of this class, [C10H22O5N]+, at *m*/*z* 236.1 corresponding to the combined loss of both fatty acids as keto derivatives (R1CO + R2CO) (Figure 7A). We also detected the characteristic ion [C7H14O2N]+ at *m*/*z* 144.1 consistent with the loss of glycerol and both fatty acids [35].

The fatty acyl composition was deducted by the loss of fatty acyl chains as acid (-RCOOH) and ketene (-R=C=O) derivatives. In the DGTS spectra, we detected a pair of fragment ions at *m*/*z* 456.3 and 474.3 corresponding to the loss of fatty acyl chains as acid and keto derivatives, respectively. This fragmentation closely matched the molecules DGTS (34:0; 16:0–18:0) and DGTS (32:0) containing two hexadecanoic (C16:0) acids (Figure 7B and [36]).

**Figure 8:**
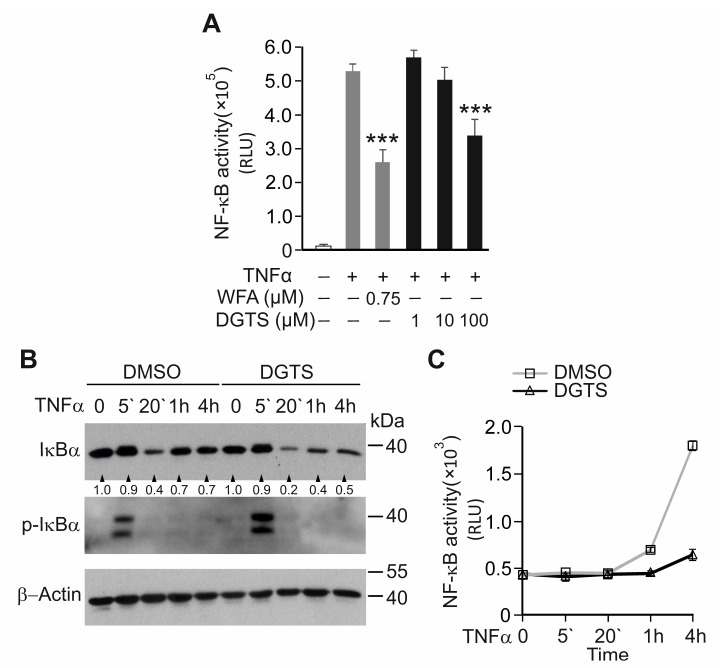
The betaine lipid DGTS (32:0) acts as a bioactive compound inhibiting TNFα-induced NF-κB activity. (**A**) Dose-specific inhibition of NF-κB-dependent luciferase activity by DGTS in TNFα-stimulated HaCaT cells. Cells were incubated with the indicated concentrations of DGTS for 1 h, stimulated with 0.75 ng mL^−1^ TNFα for 6 h, and analyzed by luciferase assay. *** *p* < 0.001 vs. samples treated with DMSO and TNFα. (**B**) Effects of DGTS on the degradation and re-synthesis of IκBα in response to TNFα. NF-κB-reporter HaCaT cells were treated with TNFα as described in Figure 3B in the presence or absence of 100 µM DGTS. Cell lysates were analyzed by immunoblotting using IκBα, phospho-IκBα and β-Actin-specific antibodies. The intensity of the bands was expressed as a fold change relative to the control. (**C**) NF-κB reporter activities were measured in cell lysates from (**B**). Values are means ± SD of at least three measurements.

To investigate whether the 1,2-dihexadecanoyl-sn-glycero-3-O-(N,N,N-trimethyl)-homoserine (DGTS (32:0)) identified in NAE_2022C affected the TNFα-induced NF-κB response, we treated NF-κB reporter HaCaT cells with increasing DGTS concentrations (Figure 8A). DGTS significantly reduced NF-κB-luciferase activity in the TNFα-stimulated HaCaT cells at a concentration of 100 µM without compromising cell viability. Next, we tested whether DGTS could reiterate the effect of NAE_2022C on the IκBα degradation induced by TNFα. As shown in Figure 8B,C, DGTS inhibited an NF-κB-dependent increase in luciferase activity, observed already after 1 h of TNFα stimulation, but did not prevent the phosphorylation and degradation of IκBα. Consistent with the inhibition of NF-κB, the re-synthesis of IκBα at the late time points of TNFα stimulation was markedly repressed by DGTS. These findings suggest that DGTS likely affects NF-κB activation downstream of the IKK complex, or through indirect inhibitory mechanisms. Indeed, we showed that, in contrast to NAE_2022C or its active fraction (F7 + F8), DGTS alone did not inhibit the activity of IKKβ or the phosphorylation of the IκBα protein in the in vitro kinase assay (Figure S2). Thus, we identified DGTS (32:0) as an active component of NAE_2022C with anti-inflammatory activity, excluding its direct role in the inhibition of the IKK complex by NAE_2022C.

## 4. Discussion

Terrestrial microalgae are recognized as efficient producers of secondary metabolites with different bioactivities [15,22]. In the present study, we screened a number of algae strains from the unique ASIB 505 algae collection for their potential to synthesise and accumulate metabolites with anti-inflammatory properties. To improve the manifold of metabolites, we used a two-stage cultivation strategy [37,38]. In the two-stage cultivation, microalgae were initially grown under standard, nutrient-rich conditions to obtain maximum biomass and, thereafter, various stress conditions were applied to trigger the accumulation of metabolites. The screening of crude algae extracts allowed us to identify a unicellular green algae *C. zofingiensis*, revealing unique anti-inflammatory activities in TNFα-stimulated human keratinocyte HaCaT cells and in a human epidermis (epiCS) model.

TNFα is a major pro-inflammatory cytokine that primarily acts through the activation of the NF-κB pathway downstream of TNFR1, leading to the expression and release of inflammatory mediators [39]. Therefore, the suppression of NF-κB may be an attractive strategy for the development of anti-inflammatory medication [40]. In this study, we found that polar and, to a larger extent, nonpolar extracts obtained from *C. zofingiensis* selectively suppressed TNFα-stimulated NF-κB and ERK1/2 activities without inhibiting JNK and p38 signaling in HaCaT cells. NAE_2022C efficiently prevented the phosphorylation and degradation of IκBα and consequently blocked the nuclear accumulation and transcriptional activity of NF-κB. The TNFR/NF-κB pathway analysis revealed that NAE_2022C acted downstream of TAK1 by directly targeting IKKβ kinase and interfering with the IKK complex functions (Figure 4B). The ability of NAE_2022C to selectively inhibit IKK-mediated responses without targeting other signaling branches in the TNFR pathway may provide an important advantage for therapeutic applications, e.g., by reducing immunosuppressive effects frequently caused by broad-range NF-κB-inhibiting agents [26,41]. Indeed, the JNK and p38 pathways have been implicated at later stages during immune responses by regulating expression of immune modulators or being involved in T cell differentiation [42,43]. Thus, the preservation of these pathways upon the treatment of cells with NAE_2022C could be regarded as beneficial. Furthermore, we demonstrated that NAE_2022C acts in a stimulus-specific manner, showing a high potential to inhibit TNFα- but not UVB-mediated NF-κB responses. Different signaling routes utilized by those stimuli could explain this. It has been shown that UV light activates NF-κB via p38-CK2 and/or JNK-β-TrCP axes by promoting the IKK-independent degradation of IκBα [44,45]. Consistently, the phosphorylation of JNK and p38 was not suppressed but enhanced in NAE_2022C-treated cells. The enhanced phosphorylation of JNK is likely to be caused by the NAE_2022C-mediated decrease in basal and TNFα-induced NF-κB activity required for the suppression of JNK signaling [46,47]. The mechanism by which NAE_2022C augments p38 responses remains unclear. The described properties distinguish NAE_2022C from common anti-inflammatory NF-κB-inhibiting drugs, such as dexamethasone and withaferin A. These compounds impede NF-κB activation in response to both TNFα and UV radiation by having broader targeting effects and/or acting downstream in the pathway. WFA, which is a major constituent of the plant *Withania somnifera*, prevents IκBα degradation by acting as a proteasomal inhibitor [27,48] or by targeting the IKK complex [49,50]; it also inhibits p65/RelA and p50 dimerization and the DNA-binding activity of NF-κB [28,51]. Glucocorticoids interfere with NF-κB through direct and indirect mechanisms acting primarily at the transcriptional level [26,52]. Indirectly, glucocorticoids induce the transcription and synthesis of IκBα, enhancing the retention of NF-κB in the cytoplasm and inhibiting its activation [53,54]. However, under certain conditions, glucocorticoids can directly inhibit activated NF-κB via competition between p65/RelA and the glucocorticoid receptor for a limited nuclear pool of co-activators [55,56]. Hence, we demonstrated that NAE_2022C is a potent NF-κB suppressor, exhibiting a rather unique and highly specific mode of action.

The bioactivity of NAE_2022C was further verified in a reconstituted human epidermis (epiCS) that resembled the organization of fully differentiated tissue with a base membrane, proliferating keratinocytes, and a stratum corneum [57]. Our results showed that NAE_2022C suppressed the secretion of pro-inflammatory cytokine TNFα, and Th1- and Th2-related chemokines, supporting its anti-inflammatory and potentially immune modulating activities in a physiologically relevant human epidermis model. However, follow-up studies in animal models of inflammatory diseases, e.g., in a mouse model of atopic dermatitis [58] or chemically induced colitis [59], will be mandatory to provide more in vivo knowledge on the suitability of NAE_2022C and its individual components for general therapeutic application.

To identify the active compounds responsible for specific anti-inflammatory effects, bioassay-guided fractionation of the crude NAE_2022C extract was performed. UHPLC-qTOF-MS/MS analysis identified several diverse compounds, including betaine lipid DGTS and its lyso form LDGTS, phenolic compounds (5-phenylvaleric acid and theophylline), and primary fatty acid amide (oleamide) as major constituents of the determined active fraction (F7 + F8). Various polar lipids have previously been reported with biological activity, for example, some species of DGTS, SQDG, MGDG and DGDG [60]. In particular, DGTS (40:10) acts as an anti-inflammatory agent inhibiting nitric oxide (NO) production in RAW264.7 macrophage cells through the downregulation of inducible nitric oxide synthase (iNOS) [61]. Here, we showed that DGTS (32:0), identified in NAE_2022C, efficiently inhibited TNFα-induced NF-κB activation in human keratinocyte cell line without compromising cell viability. The polar lipid content in *C. zofingiensis* can reach up to 10% of its dry weight when algae are grown under nitrogen deprivation, and their methanol-based extracts are normally enriched with these components [62,63]. However, the anti-inflammatory effects of DGTS and its derivatives need careful interpretation, as the actual amounts of this lipid in algae extracts might be lower than doses used in the in vivo studies. We also found that DGTS inhibited NF-κB by utilizing mechanisms independent of IKKβ inhibition, acting downstream or indirectly. We, therefore, conclude that, in addition to DGTS, other components in the active NAE_2022C fraction are required to provide a full spectrum of inhibitory effects, including the inhibition of the IKK complex. Literature studies show that some phenolic compounds, e.g., flavonoids and phenolic acids, have an anti-inflammatory effect when used as dietary supplements [64]. Although there is no direct evidence for the anti-inflammatory activity of 5-phenylvaleric acid, theophylline has been reported as a systemic inflammation-reducing agent with modest phosphodiesterase (PDE)- and NF-κB-inhibiting activities [65,66,67]. It remains to be investigated, however, to what extent these phenolic compounds contribute to the anti-inflammatory effects of NAE_2022C. In the active NAE_2022C fraction, oleamide was identified as a less abundant component when compared to others. Nevertheless, oleamide has attracted our attention as a known anti-inflammatory metabolite which can be synthesized de novo in the mammalian nervous system, and has been detected in human plasma [68]. Moon S.-M. et al. reported that oleamide reduced the expression of inflammatory mediators such as iNOS, COX-2, and pro-inflammatory cytokines by inhibiting ERK1/2 and JNK phosphorylation and NF-κB activity in LPS-induced RAW264.7 macrophages [69]. Based on our findings and reported facts, it becomes apparent that the combination of several metabolites rather than the activity of a single compound specifies the anti-inflammatory properties of NAE_2022C. Further studies are needed to elucidate the specific inhibitory mechanisms and cellular targets of each of the identified active metabolites and to better understand their interactions as components of a complex anti-inflammatory compound.

Together, our study identified *C. zofingiensis* as a natural source of various anti-inflammatory metabolites synthesized under the specified cultivation conditions. Crude *C. zofingiensis* extracts or their specific fractions can be used as complex formulations to specifically inhibit NF-κB-dependent inflammatory responses. These findings may provide a basis for the identification and development of attractive therapeutic candidates with fewer adverse effects.

## Data Availability

The mass spectrometry data are available on request from the corresponding authors. The data are not publicly available due to a patent application request.

## Supplementary Materials

The following supporting information can be downloaded at: https://www.mdpi.com/article/10.3390/cells11091407/s1, Figure S1: Validation of primary hits. Dose-dependent effects on NF-κB luciferase reporter activities of the selected algal extracts in TNFα-stimulated HaCaT cells. NF-κB activity in DMSO-treated cells is normalized to 100%. Each curve represents an independent experiment. Values are mean ± SD of technical replicates (*n* = 3); Ctr, control (DMSO + TNFα); Figure S2: The active NAE_2022C fraction (F7 + F8), but not DGTS, directly inhibits IKKβ kinase activity. IKKβ activity was analyzed by in vitro kinase assay using recombinant HIS-IκBα and [γ-^32^P]ATP in the presence of DMSO, (F7 + F8) fraction or DGTS (1, 10, 100 µM). The reactions were run on SDS-PAGE and analyzed by autoradiography; IgHC, immunoglobulin heavy chain; IgLC, immunoglobulin light chain; Figure S3: Original images. Dashed line designates the cropped area; Table S1: List of algae strains selected from the ASIB 505 collection; Table S2: Cytotoxicity of selected algae extracts; Table S3: UHPLC-qTOF-MS/MS analysis of NAE_2022C active fraction (F7 + F8).
