# Supplementary material for: Anti-Inflammatory Extract from Soil Algae Chromochloris zofingiensis Targeting TNFR/NF-κB Signaling at Different Levels
Source: Cells. 2022 Apr 21;11(9):1407. doi: 10.3390/cells11091407 (PMC9101025; doi:10.3390/cells11091407)
Supplement: Supplementary file 1 [file cells-11-01407-s001.zip › cells-1627771-supplementary.pdf]

Supplementary information

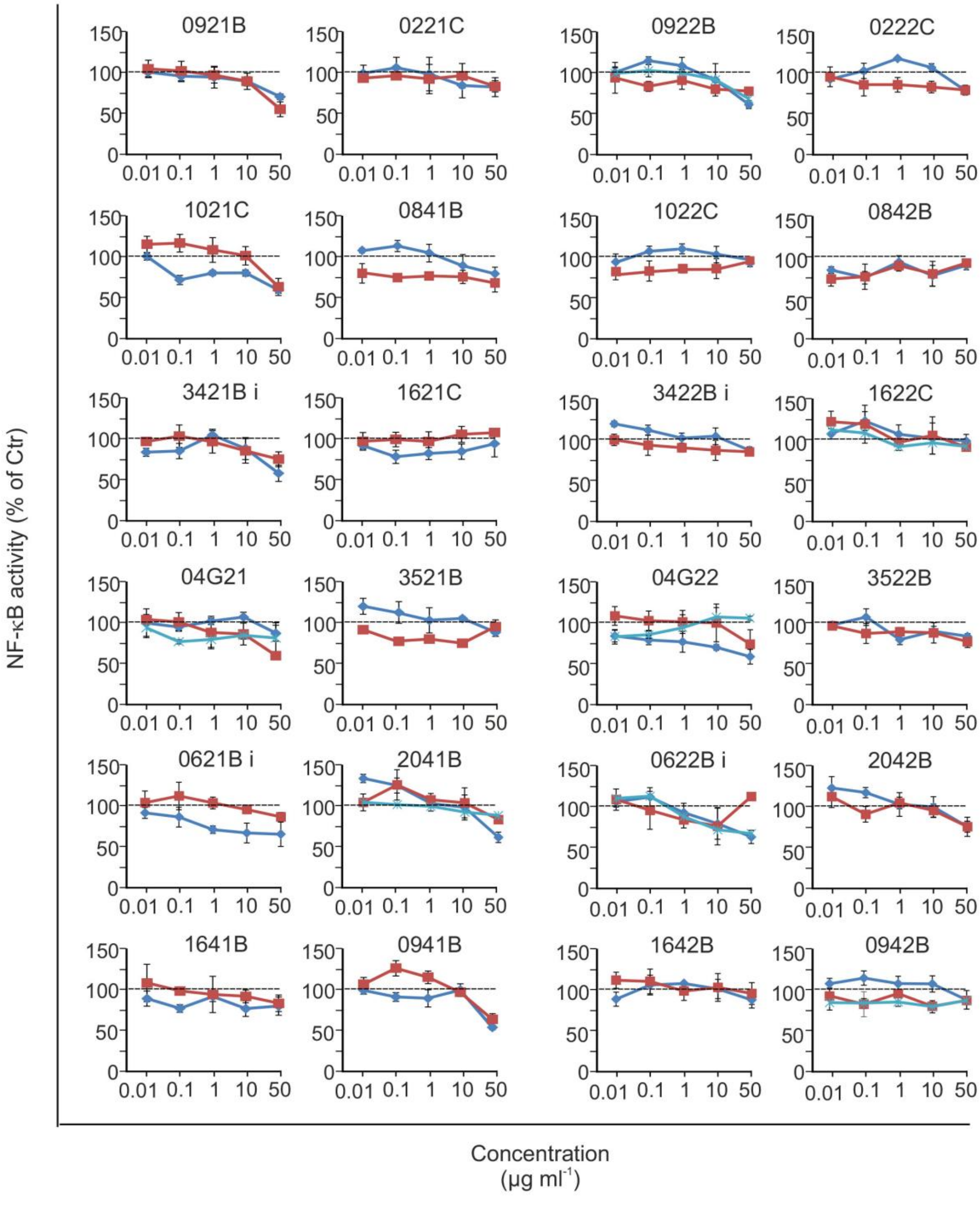

Figure S1. Validation of primary hits. Dose-dependent effects on NF-κB luciferase reporter activities of the selected algal extracts in TNFα-stimulated HaCaT cells. NF-κB activity in DMSO-treated cells is normalized to 100%. Each curve represents an independent experiment. Values are mean ± SD of technical replicates (*n* = 3); Ctr, control (DMSO + TNFα).

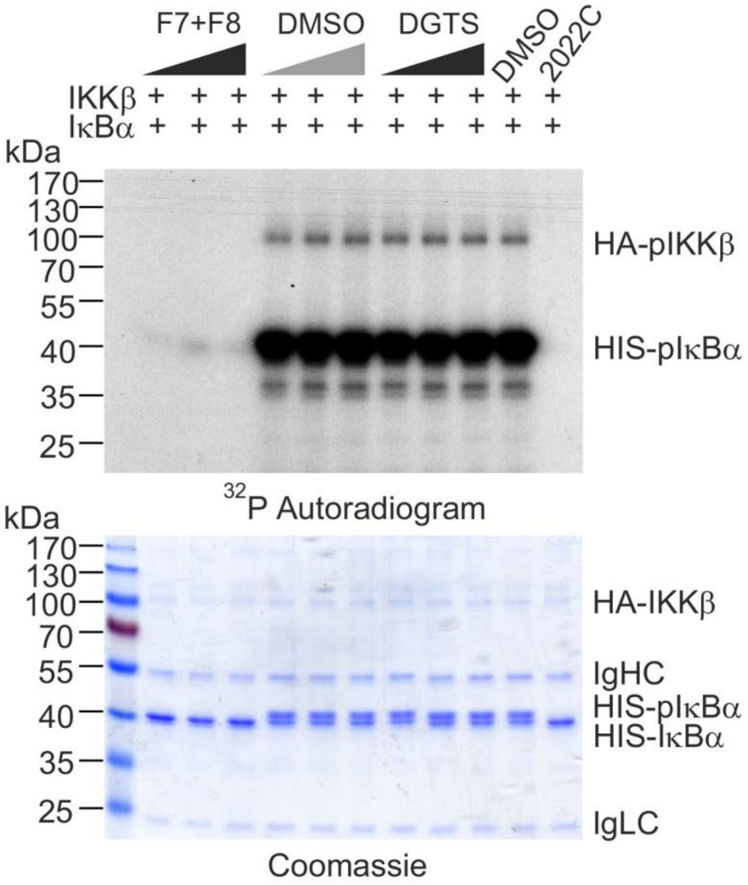

Figure S2. The active NAE_2022C fraction (F7+F8), but not DGTS, directly inhibits IKKβ kinase activity. IKKβ activity was analyzed by *in vitro* kinase assay using recombinant HIS-IκBα and [ϒ-^{32}P]ATP in the presence of DMSO, (F7+F8) fraction or DGTS (1, 10, 100 μM). The reactions were run on SDS-PAGE and analyzed by autoradiography; IgHC, immunoglobulin heavy chain; IgLC, immunoglobulin light chain.

Figure 3B
WB: anti-IκBα

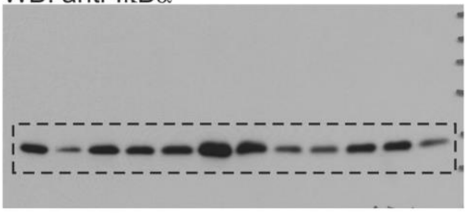

Figure 3B
WB: anti-β-Actin

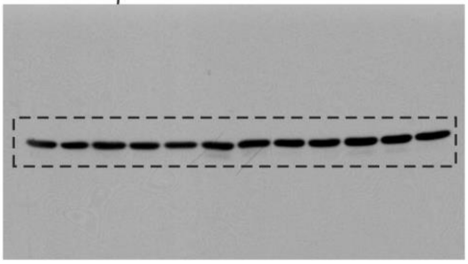

Figure 3B
WB: anti-pIκBα

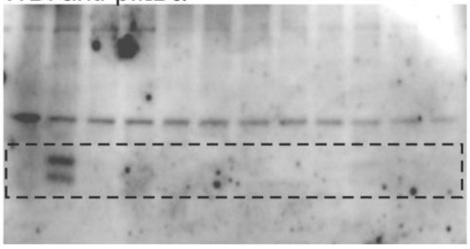

Figure 3B
WB: anti-β-Actin

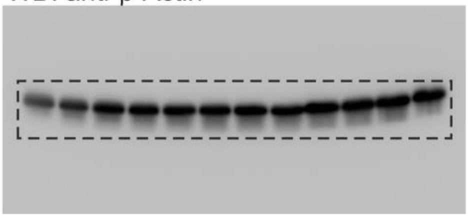

Figure 4A
WB: anti-IκBα

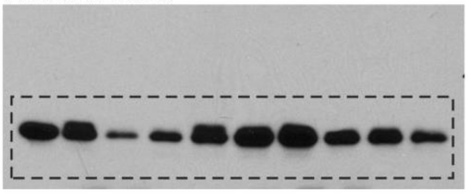

Figure 4A
WB: anti-pIκBα

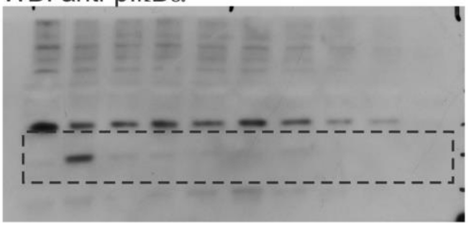

Figure 4A
WB: anti-pp38

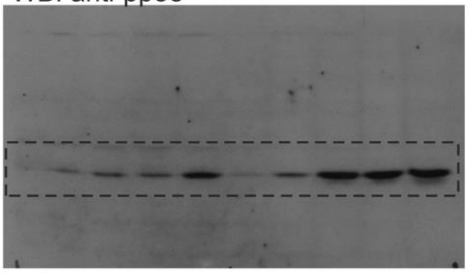

Figure 4A
WB: anti-β-Actin

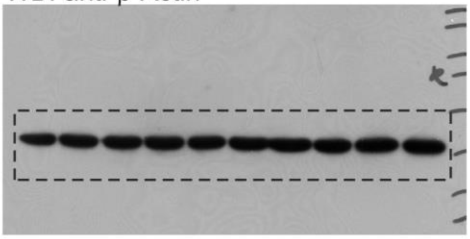

Figure 4A
WB: anti-pJNK

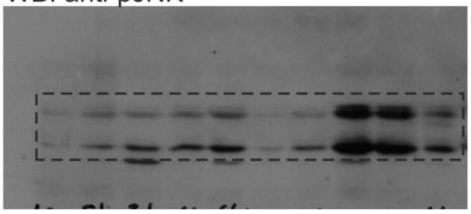

Figure 4A
WB: anti-β-Actin

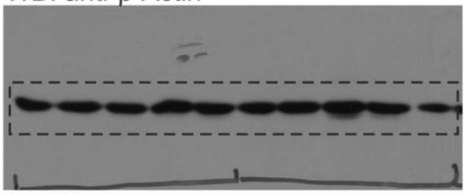

Figure 4A
WB: anti-pERK1/2

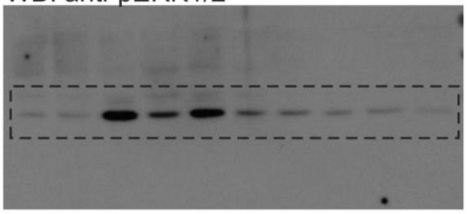

Figure 4A
WB: anti-β-Actin

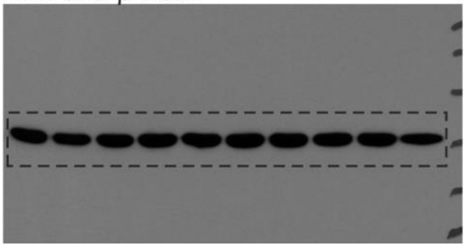

Figure S3. Original images. *Dashed line designates the cropped area.*

Table S1. List of algae strains selected from the ASIB 505 collection.

Algae Species	Collection ID	Growth in 3N-BBM	PAE ID	NAE ID
Subphylum: Chlorophytina	IB459	+++	0121<br>0141	0122<br>0142
Coccomyxa subellipsoidea	BS110	+++	0221<br>0241	0222<br>0242
Klebsormidium subellipsoidea or Interphylum subellipsoidea	IB505	+++	0321	0322
Chromochloris zofingiensis<br>*Order: Sphaeropleales*	V46	+	04G21<br>04G51	04G22<br>04G52
Xanthophyceae	V168	+++	0521	0522
n.a.	IB514	+++	0621<br>0641	0622<br>0642
Coccomyxa subellipsoidea	T3	+++	0721	0722
Pseudochlorella subsphaerica<br>*Family: Prasiolaceae*	BS775	+++	0821<br>0841	0822<br>0842
Coccomyxa subellipsoidea<br>*Family: Coccomyxaceae*	IB273<br>(PUFA)	+++	0921<br>0941	0922<br>0942
Neocystis brevis<br>*Subphylum Chlorophytina*	IB410	+++	1021	1022
Bracteacoccus subellipsoidea	V103<br>V219	+++	1121<br>1221	1122<br>1222
Ectocarpus siliculosus<br>*Family: Ectocarpaceae*	V50	+++	1321	1322
Pseudostichococcus subellipsoidea, Phylum: Chlorophyta	SAG 379 1c	+++	1411	1412
Trebouxiophyceae subellipsoidea	IB423	+++	1521	1522
Chromochloris zofingiensis<br>*Order: Sphaeropleales*	IB408	+++	1621<br>1641	1622<br>1642
Chlorella saccharophila	CV. Vulgaris	+++	1721<br>1741	1722<br>1742
Diplosphaera subellipsoidea, Subphylum: Chlorophytina	T61	++	1821	1822
Bracteococcus subellipsoidea	V195	+	1921	1922
Chromochloris zofingiensis<br>*Subphylum: Chlorophytina*	V142	+++	2021<br>2041	2022<br>2042
Edaphochlorella mirabilis	IB407<br>(PUFA)	+++	2121<br>2131	2122<br>2132

			2141	2142
n.a.	V6	+++	2221	2222
Bracteacoccus subellipsoidea	T87	+++	2321	2322
Scenedesmus subellipsoidea	V21	+	2411	2412
Dictyococcus varians<br>*Order: Sphaeropleales*	V204	+++	2521<br>2551	2522<br>2552
Coccomyxa subellipsoidea	IB256	+	2611<br>2641	2612<br>2642
Coccomyxa subellipsoidea	V199	+++	2721	2722
Muriella subellipsoidea	BS319	+++	2811	2812
n.a.	V24	+	2911	2912
Ettila texensis	SAG 79.80	+++	3021	3022
Bracteacoccus subellipsoidea	V208	+	3111	3112
n.a.	CCALA 496	++	3221	3222
Coelastrella subellipsoidea Subphylum Chlorophytina	CH	+	3321	3322
Nostoc subellipsoidea	BS363	+++	3421	3422
Bracteacoccus subellipsoidea	V208	+++	3521	3522
Vischeria helvetica<br>*Order: Eustigmatales*	V39	+	3611	3612

Table S2. Cytotoxicity of selected algae extracts

Extract ID	Cell viability (% of control)			
	0.1	1	10	100
	(μg ml^{-1})			
**0121B i**	100 ± 6	102 ± 7	102 ± 9	98 ± 9
**0141B**	102 ± 3	89 ± 8	96 ± 2	103 ± 2
**0221C**	102 ± 5	103 ± 5	102 ± 4	93 ± 1
**0241C**	101 ± 1	101 ± 2	99 ± 2	99 ± 6
**0321B**	101 ± 2	102 ± 1	102 ± 1	97 ± 1
**04G21**	105 ± 8	109 ± 11	106 ± 8	92 ± 1
**04G51**	100 ± 1	102 ± 1	101 ± 2	88 ± 5
**0521B**	102 ± 1	99 ± 1	99 ± 2	92 ± 4
**0621B i**	104 ± 7	102 ± 1	105 ± 7	86 ± 4
**0641B**	102 ± 6	102 ± 2	101 ± 3	97 ± 2
**0721B**	107 ± 6	109 ± 4	111 ± 4	110 ± 3
**0821B i**	104 ± 2	103 ± 1	104 ± 1	78 ± 10
**0841B**	102 ± 4	100 ± 2	97 ± 1	76 ± 4
**0921B**	101 ± 2	104 ± 4	102 ± 3	96 ± 4
**0941B**	101 ± 3	100 ± 4	99 ± 5	97 ± 2
**1021C**	101 ± 2	102 ± 5	99 ± 2	68 ± 2
**1121C**	100 ± 5	98 ± 2	96 ± 3	86 ± 3
**1221B**	97 ± 1	98 ± 3	96 ± 3	90 ± 2
**1321B**	102 ± 2	102 ± 1	101 ± 4	91 ± 3
**1411B**	103 ± 5	102 ± 6	102 ± 2	98 ± 2
**1521B**	103 ± 3	103 ± 2	104 ± 4	101 ± 2
**1621C**	103 ± 3	103 ± 4	104 ± 2	55 ± 2
**1641B**	102 ± 3	102 ± 1	100 ± 2	97 ± 2
**1721C**	102 ± 2	103 ± 3	103 ± 4	95 ± 2
**1741B**	100 ± 4	104 ± 3	101 ± 4	99 ± 1
**1821B**	102 ± 5	101 ± 3	100 ± 1	89 ± 3
**1921C**	102 ± 3	101 ± 2	102 ± 2	92 ± 1
**2021C**	101 ± 3	100 ± 2	102 ± 3	93 ± 3
**2041B**	102 ± 2	100 ± 1	101 ± 3	99 ± 2
**2121B i**	102 ± 1	101 ± 2	101 ± 1	94 ± 1
**2131B**	100 ± 4	103 ± 3	98 ± 2	89 ± 2
**2141B**	105 ± 2	102 ± 2	100 ± 6	90 ± 7
**2221B**	102 ± 1	99 ± 1	98 ± 4	90 ± 6
**2321C**	100 ± 4	102 ± 2	98 ± 3	90 ± 2
**2411B**	104 ± 2	103 ± 1	98 ± 3	96 ± 3
**2521C**	107 ± 3	103 ± 5	109 ± 4	96 ± 7
**2551B**	99 ± 1	94 ± 6	95 ± 3	89 ± 4

**2611B**	108 ± 3	106 ± 3	108 ± 2	97 ± 5
**2641B**	100 ± 1	100 ± 4	101 ± 3	99 ± 2
**2721B**	105 ± 2	110 ± 4	105 ± 5	95 ± 3
**2811C**	101 ± 3	103 ± 2	102 ± 4	99 ± 4
**2911B**	100 ± 4	102 ± 1	102 ± 5	99 ± 1
**3021B**	102 ± 3	103 ± 1	105 ± 4	92 ± 3
**3111B**	108 ± 2	108 ±4	109 ± 2	104 ± 3
**3221B**	101 ± 4	102 ± 3	103 ± 4	98 ± 3
**3321C**	105 ± 2	106 ± 2	102 ± 3	100 ± 2
**3421B i**	103 ± 3	102 ± 2	99 ± 2	99 ± 4
**3521B**	103 ± 2	100 ± 4	102 ± 4	98 ± 4
**3611B**	92 ± 2	92 ± 2	95 ± 2	72 ± 3
**0122B i**	98 ± 4	99 ± 4	96 ± 5	94 ± 2
**0142B**	103 ± 3	103 ± 5	104 ± 2	95 ± 3
**0222C**	101 ± 5	102 ± 3	103 ± 3	102 ± 4
**0242C**	94 ± 3	97 ± 2	94 ± 2	96 ± 4
**0322B**	97 ± 4	102 ± 2	99 ± 5	92 ± 5
**04G22**	105 ± 4	99 ± 3	105 ± 2	98 ± 3
**04G52**	103 ± 5	103 ± 7	99± 6	92 ± 3
**0522B**	102 ± 2	103 ± 3	104 ± 5	93 ± 2
**0622B i**	104 ± 3	101 ± 2	98 ± 2	94 ± 1
**0642B**	103 ± 1	102 ± 3	100 ± 4	101 ± 2
**0722B**	101 ± 2	100 ± 1	101 ± 2	92 ± 3
**0822B i**	102 ± 3	110 ± 5	106 ± 4	104 ± 2
**0842B**	95 ± 4	94 ± 4	97 ± 3	95 ± 2
**0922B**	95 ± 5	96 ± 2	98 ± 2	80 ± 3
**0942B**	91 ± 6	100 ± 4	99 ± 5	89 ± 4
**1022C**	93 ± 2	98 ± 4	101 ± 1	100 ± 4
**1122C**	102 ± 2	101 ± 1	104 ± 2	103 ± 2
**1222B**	100 ± 2	103 ± 2	104 ± 3	101 ± 3
**1322B**	99 ± 1	104 ± 1	103 ± 3	100 ± 4
**1412B**	98 ± 5	98 ± 3	103 ± 4	89 ± 3
**1522B**	100 ± 3	102 ± 2	104 ± 1	102 ± 2
**1622C**	106 ± 1	108 ± 2	108 ± 1	102 ± 2
**1642B**	102 ± 3	98 ± 2	103 ± 4	96 ± 7
**1722C**	105 ± 1	108 ± 2	107 ± 1	109 ± 3
**1742B**	100 ± 1	101 ± 5	101 ± 4	95 ± 5
**1822B**	105 ± 4	102 ± 4	103 ± 5	99 ± 2
**1922C**	100 ± 2	101 ± 3	100 ± 1	96 ± 2
**2022C**	100 ± 1	101 ± 3	103 ± 2	99 ± 2
**2042B**	100 ± 4	99 ± 3	101 ± 2	97 ± 5
**2122B i**	100 ± 1	102 ± 3	105 ± 2	104 ± 3

**2132B**	100 ± 4	104 ± 3	103 ± 5	105 ± 3
**2142B**	100 ± 1	101 ± 3	99 ± 2	95 ± 2
**2222B**	102 ± 4	98 ± 4	90 ± 2	89 ± 4
**2322C**	100 ± 4	104 ± 3	103 ± 5	105 ± 3
**2412B**	102 ± 3	99 ± 1	101 ± 3	98 ± 5
**2522C**	101 ± 2	99 ± 3	102 ± 2	94 ± 2
**2552B**	102 ± 1	101 ± 3	103 ± 2	98 ± 4
**2612B**	93 ± 5	95 ± 5	95 ± 4	98 ± 7
**2642B**	96 ± 3	95 ± 2	96 ± 4	97 ± 3
**2722B**	95 ± 6	96 ± 4	100 ± 2	75 ± 4
**2812C**	97 ± 2	96 ± 2	99 ± 1	94 ± 2
**2912B**	100 ± 5	102 ± 6	99 ± 1	95 ± 3
**3022B**	97 ± 1	95 ± 2	98 ± 2	97 ± 2
**3112B**	102 ± 3	105 ± 2	98 ± 3	94 ± 7
**3222B**	91 ± 2	89 ± 2	96 ± 2	103 ± 2
**3322C**	100 ± 2	101 ± 3	99 ± 1	94 ± 2
**3422B i**	100 ± 3	98 ± 5	99 ± 4	98 ± 2
**3522B**	99 ± 3	102 ± 3	98 ± 7	95 ± 4
**3612B**	94 ± 2	94 ± 2	95 ± 3	94 ± 4

Table S3. UHPLC-qTOF-MS/MS analysis of NAE_2022C active fraction (F7+F8)

Compound	Molecular formula	RT [min]	Calculated m/z $[M+H]^+$	Experimental m/z $[M+H]^+$	Δm/z [ppm]	Δm/z [mDa]	Structure
LDGTS 16:0	$C_{26}H_{51}NO_6$	6.28	473.373	474.381	3.888	1.844	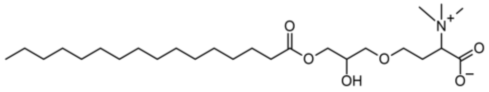
5-Phenylvaleric acid	$C_{11}H_{14}O_2$	9.16	178.099	179.106	4.849	0.869	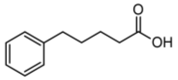
Oleamide	$C_{18}H_{35}NO$	10.08	281.273	282.280	1.954	0.552	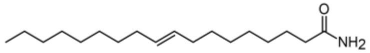
Theophylline	$C_7H_8N_4O_2$	10.57	180.081	181.088	0.995	0.194	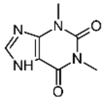
DGTS 32:0; DGTS 16:0-16:0	$C_{42}H_{81}NO_7$	11.43	712.612	712.619	0.768	0.424	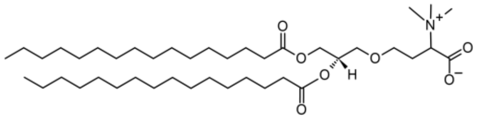
DGTS 34:0; DGTS 16:0-18:0	$C_{44}H_{85}NO_7$	11.41	739.636	740.643	3.911	2.896	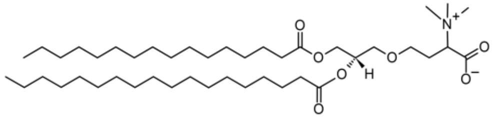
